# Immunization with a Synthetic *Helicobacter pylori* Peptide Induces Secretory IgA Antibodies and Protects Mice against Infection

**DOI:** 10.1155/2019/8595487

**Published:** 2019-04-01

**Authors:** David Espinosa-Ramos, Diana Caballero-Hernández, Ricardo Gomez-Flores, Armando Trejo-Chávez, Luis Jerónimo Pérez-Limón, Myriam Angélica de la Garza-Ramos, Reyes Tamez-Guerra, Patricia Tamez-Guerra, Cristina Rodriguez-Padilla

**Affiliations:** ^1^Universidad Autónoma de Nuevo León, Facultad de Ciencias Biológicas, Departamento de Microbiología e Inmunología, San Nicolás de los Garza, NL. C.P. 66450, Mexico; ^2^Universidad Autónoma de Nuevo León, Facultad de Medicina Veterinaria y Zootecnia, Departamento de Patobiología, Campus de Ciencias Agropecuarias, Escobedo, NL. C.P. 66050, Mexico; ^3^Universidad Autónoma de Nuevo León, Facultad de Odontología y Centro de Investigación y Desarrollo en Ciencias de la Salud, Unidad de Odontología Integral y Especialidades, Av. Dr. Aguirre Pequeño y Silao S/N, Monterrey, NL. C.P. 64460, Mexico

## Abstract

*Helicobacter pylori* is a spiral Gram-negative bacterium associated with inflammation of the gastric mucosa, peptic ulcer, and gastric adenocarcinoma, whose treatment has failed due to antibiotic resistance and side effects. Furthermore, because there are no vaccines effective against *H. pylori*, an appropriate vaccine design targeting conserved/essential genes must be identified. In the present study, a *H. pylori* 50–52 kDa immunogen-derived peptide antigen with the sequence Met-Val-Thr-Leu-Ile-Asn-Asn-Glu (MVTLINNE) was used to immunize against *H. pylori* infection. For this, mice received an intraperitoneal injection of 100 *μ*g of *H. pylori* peptide on the first week, followed by two weekly subcutaneous reinforcements and further 10^9^ bacteria administration in the drinking water for 3 weeks. Thymic cells proliferative responses to concanavalin A, serum levels of IL-2, IL-4, IL-6, IL-10, IL-17, IFN-*γ*, and TNF-*α* cytokines, and IgG1, IgG2a, IgG2b, IgG3 IgM, and IgA immunoglobulins were evaluated. Significant (*p* < 0.05) increases on lymphoproliferation and spleen weights after immunization were observed. In contrast, infection significantly (*p* < 0.05) decreased lymphoproliferation, which was recovered in immunized mice. In addition, levels of serum TH1 and TH2 cytokines were not altered after immunization, except for the significant increase in IL-6 production in immunized and/or infected animals. Moreover, immunization correlated with plasma secretory IgA and IgG, whereas infection alone only elicited IgM antibodies. Peptide immunization protected 100% of mice against virulent *H. pylori*. MVTLINNE peptide deserves further research as an approach to the prophylaxis of *H. pylori* infection.

## 1. Introduction

*H. pylori* is a Gram-negative spiral-shaped bacterium that represents the main factor for the development of human chronic gastritis, duodenal ulcer, and gastric adenocarcinoma [1–3]. Despite the decrease in the incidence of gastric carcinoma due to *H. pylori* in recent years, this disease is still the most common cause of death from cancer worldwide. In fact, it is the fourth cause of cancer cases per year, according to a 2000 report, with 945,000 new cases [1]. In developed countries, 70 to 90% of the population acquires the infection before 10 years of age and its routes of transmission are oral-oral or fecal-oral, but iatrogenia may be also involved, when performing endoscopy with a contaminated tube [4, 5]. In addition to surgery, which includes partial gastrectomy, a wide variety of antibiotics have been proposed for the treatment of gastric ulcer accepted by the Food and Drug Administration, namely, the use of bismuth subsalicylate, metronidazole, and tetracycline, along with an antacid agent; however, this regimen can cause systemic damage, such as pseudomembranous colitis in 11% and vaginal candidiasis in excess of 10% in women under treatment [4]. Commonly, the first-line treatment consists of a 7 to 10 days regimen with a proton-pump inhibitor plus amoxicillin and clarithromycin [6]. However, antibiotics are affected by increasing levels of resistance [7, 8]. For instance, clarithromycin resistance has recently been reported in 26%, 27.2%, and 25% of patients infected by *H. pylori* in France, Spain, and Italy, respectively [9]. However, in developing countries, particularly in Mexico, resistance reaches 28.2% [10]. It has recognized that infection is strongly associated with the socioeconomic and sociodemographic conditions of the population where the variation of *H. pylori* virulence-associated genotypes could favor the development of gastrointestinal tract pathologies in infected patients [11]. Because of this, there has been an increasing interest in the development of vaccines as a prophylaxis to *H. pylori* infection [12].

In experimental studies, it has been observed that the use of 52 kDa *H. pylori* membrane peptide as a vaccine has been effective to immunize against the development of gastric ulcer when used in murine models. However, the isolation and purification of such a protein presents important challenges; therefore, the use of synthetic peptides designed from immunogenic proteins has become an alternative for diagnosis and prophylaxis. Since *H. pylori* causes a superficial infection of the gastric tissue, the main immunity mediators are secretory lgA antibodies, which are the objectives of active oral vaccination [13]. Immunized animals produce specific serum IgG and IgA, and intestinal and salivary IgA, and, after challenge, a gastric cellular and antibody response can be observed [14–16].

The aim of the present study was to evaluate the preventive effectiveness of vaccination with the MVTLINNE peptide, designed from a 52 kDa *H. pylori* immunogenic protein in a murine model.

## 2. Materials and Methods

### 2.1. Reagents, Cell Line, and Culture Media

Penicillin-streptomycin solution, L-glutamine, phosphate-buffered saline (PBS), and RPMI 1640 medium were obtained from Life Technologies (Grand Island, NY). Fetal bovine serum (FBS), sodium dodecyl sulfate (SDS), *N*,*N*-dimethylformamide (DMF), 3-[4,5-dimethylthiazol-2-yl]-2,5-diphenyltetrazolium bromide (MTT), and concanavalin A (Con A) were purchased from Sigma-Aldrich (St. Louis, MO). RPMI 1640 medium supplemented with 10% FBS, 1% L-glutamine, and 0.5% penicillin-streptomycin solution was referred as complete RPMI 1640 medium. *H. pylori* strain ATCC 700824 was purchased from the American Type Culture Collection (Rockville, MD) and grown on *Brucella* broth at 37°C. The strain was identified by Gram staining morphology and biochemical positive tests for catalase and urease. Extraction buffer was prepared by dissolving 20% (wt/vol) SDS at 37°C in a solution of 50% each DMF and demineralized water, and the pH was adjusted to 4.7.

### 2.2. Animals

Female BALB/c mice (20–25 g) were provided by the Bioterium of Facultad de Ciencias Biológicas at Universidad Autónoma de Nuevo León. They were kept in a pathogen- and stress-free environment at 24°C, under a light-dark cycle (light phase, 06 : 00–18 : 00 h), and given water and food *ad libitum*. All animal treatments and surgical procedures were performed in accordance with the Guide for Care and Use of Laboratory Animals by the National Institute of Health (Bethesda, MD) and approved by the University Ethics and Animal Care Committee.

### 2.3. Immunization Procedure

The immunizing peptide methionine-valine-threonine-leucine-isoleucine-asparagine-asparagine-glutamic acid (MVTLINNE) was synthesized by Genscript (Nanjing, China) with >85% purity and two modifications, acetylation at the amino terminus and amidation at the carboxyl terminus. The lyophilized peptide was stored at −20°C until immunization; for this, peptide solution was prepared at a concentration of 1 mg/mL in sterile saline. Mice were immunized by s.c. injection with the *H. pylori* peptide (100 *μ*g in 500 *μ*l of distilled water) in Sigma adjuvant (Sigma-Aldrich) (1 : 1) on day 0 and in incomplete Freünd adjuvant (1 : 1) on days 21 and 28. Mice were bled and spleens and thymuses were removed on day 91.

### 2.4. *H. pylori* Challenge

*H. pylori* was cultured on *Brucella* agar under microaerophilic conditions at 37°C in 5% O_2_, 10% CO_2_, and 85% N_2_. *H. pylori* concentration was determined by CFU counts. Seventy days after the last immunization, mice were challenged with a *H. pylori* suspension (10^9^ CFU/L) in the drinking water [17], for 21 days, after which, blood was obtained by terminal cardiac puncture, and the spleen, thymus, and stomach were aseptically removed.

### 2.5. T-Cell Proliferation Assay

T-cell proliferation was determined by a colorimetric technique using MTT [18]; 100 *μ*l thymus cell suspensions (1 × 10^7^ cells/ml) from immunized, immunized plus infected, infected, and control animals were added to flat-bottomed 96-well plates (Costar, Corning, NY), containing triplicate cultures (100 *μ*l) of RPMI 1640 medium supplemented with 5% fetal bovine serum (unstimulated control), in the presence or absence of Con A (6.25 *μ*g/ml), or MVTLINNE peptide (10 *μ*g/ml) for 48 h at 37°C in 95% air-5% CO_2_ atmosphere. After incubation for 44 h at 37°C with 5% CO_2_, MTT (0.5 mg/ml, final concentration) was added, and cultures were additionally incubated for 4 h. Cell cultures were then incubated for 16 h with extraction buffer (100 *μ*l), and optical densities, resulting from dissolved formazan crystals, were then read in a microplate reader (Bio-Tek Instruments, Inc., Winooski, VT) at 540 nm. The lymphocyte proliferation index (LPI) was calculated as follows: LPI = A540 in resident or Con A-treated cells/A540 in untreated cells.

### 2.6. Plasma Cytokine and Antibody Responses

Plasma samples were evaluated for IL-2, IL-4, IL-6, IL-10, IFN-*γ*, and TNF-*α* levels, using the mouse Th1/Th2/Th17 kit (BD Biosciences, San Jose, CA), and IgG1, IgG2a, IgG2b, IgG3 IgM, and IgA, using the BDTM cytometric bead array (CBA) mouse immunoglobulin isotyping kit, by flow cytometry (Accuri C6, BD Biosciences).

### 2.7. Mouse Gastric Tissue Histopathology

Histopathological analysis of gastric biopsies from experimental mice 21 days after infection was performed. After sacrificing, infected or immunized + infected mouse stomachs were removed and washed with sterile water. A longitudinal segment along the greater curvature from the esophagus to the stomach was used for histological examination. To determine histological alterations, tissue segments were fixed in 4% formalin solution, dehydrated, paraffin-embedded, and 3 *μ*m sections stained with hematoxylin-eosin by using routine procedures. Sections were observed in an Olympus IX71 microscope, and the image acquisition was performed in an Infinity I camera with the Infinity Capture Software (Lumenera Co., Ottawa, ON). Sections were also stained with Warthin–Starry stain for *H. pylori* detection. All histological analyses were performed blinded by an experienced veterinarian pathologist [19].

### 2.8. Statistical Analysis

The results were expressed as mean ± SEM of the response of 5 animals per treatment group, from three independent experiments. Level of significance was assessed by Student's *t*-test and ANOVA.

## 3. Results

### 3.1. Effect of Immunization and Infection on Spleen Weights

**Figure 1:**
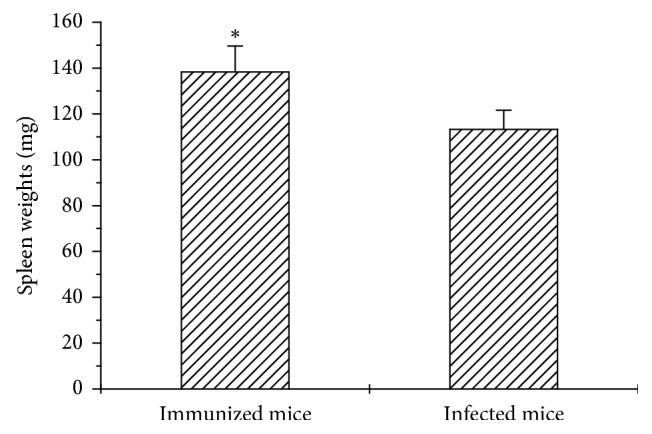
Spleen weights: spleens were removed and weighed after mice death, following immunization or *H. pylori* infection protocol, as detailed in the text. Data represent mean ± SEM of 5 animals per experimental group, from 3 independent experiments ^*∗*^*p* < 0.05.

Spleens were excised and weighed after animal's death. As seen in Figure 1, immunization significantly (*p* < 0.05) increased 37% spleen weights, in contrast to 12% nonsignificant increase in infected mice, as compared with untreated controls.

### 3.2. Effect of Immunization on Thymic Lymphocyte Proliferation

Thymus cell lymphocytes from immunized, immunized plus infected, infected, and control animals were incubated in the presence or absence of Con A or MVTLINNE peptide, and lymphoproliferative responses determined, as explained above. Con A significantly (*p* < 0.05) induced 1.2-fold increase, 1.8-fold decrease, and 1.33-fold increase in thymus lymphoproliferation from immunized, immunized plus infected, and infected, respectively, as compared with untreated control.

### 3.3. Plasma Cytokine Levels and Antibody Production

**Figure 2:**
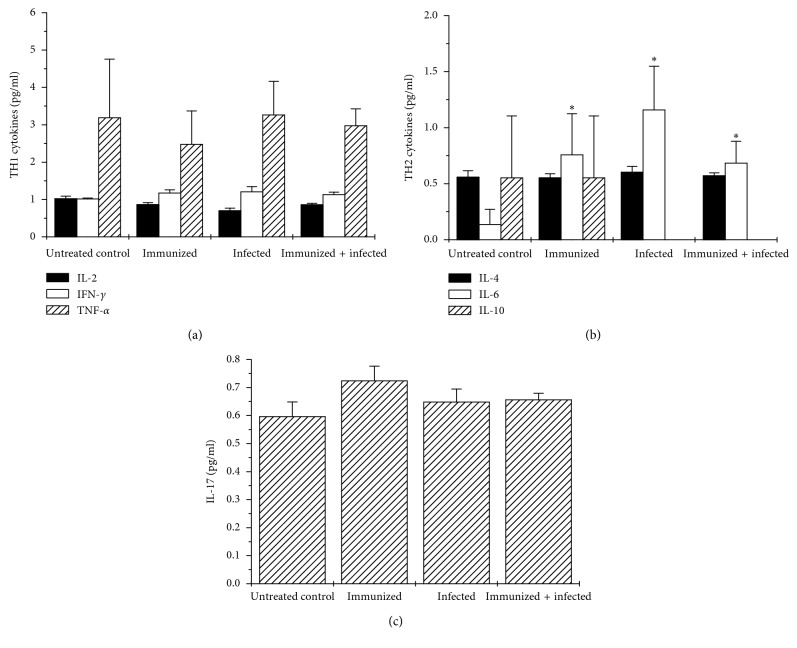
MVTLINNE peptide immunization increases plasma IL-6 levels. Plasma IL-2, IL-4, IL-6, IL-10, IL-17, IFN-*γ*, and TNF-*α* levels were measured in immunized, immunized and infected, infected, and untreated control animals, as explained in the text. Data represent mean ± SEM of 5 animals per experimental group, from 3 independent experiments. *p* < 0.05, as compared with untreated control.

As shown in Figure 2, immunization and/or infection did not alter cytokines IL-2, IL-4, IL-10, IL-17, IFN-*γ*, and TNF-*α* levels; however, IL-6 significantly (*p* < 0.05) increased, as compared with untreated control. In addition, antibody isotype observed in infected and untreated control groups was IgM, whereas immunization induced IgM, IgA, IgG1, and IgG2a antibodies; immunization and infection induced IgM, IgA, IgG1, IgG2a, IgG2b, and IgG3 antibodies (Table 1).

### 3.4. Gastric Tissue Histopathology

**Figure 3:**
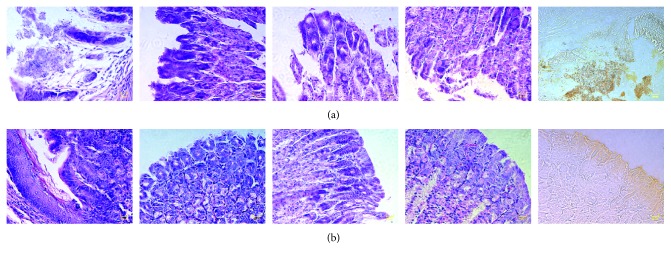
MVTLINNE peptide immunization protects mice from infection. Histological alterations and *H. pylori* presence in gastric tissue segments of infected or immunized + infected animals were determined, as explained in the text. (a) Infected animals and (b) immunized + infected animals (40x magnification).

In infected animals, at the level of the mucosa adjacent to the esophagus, a wide ulcerative area, which is composed of cellular detritus, elongated bacteria, and some spores of unicellular parasites, is observed; in addition, at the level of the submucosa, there are discrete foci of inflammatory infiltrate of mononuclear cells mainly constituted by lymphocytes and some plasma cells (Figure 3(a)). When performing the Warthin–Starry stain, elongated *H. pylori* bacteria and spores are also observed (Figure 3(a)). The diagnosis was ulcerative gastritis with presence of bacteria in gastric epithelium. In regard to immunized and infected animals, no ulcerative, inflammatory, degenerative, or neoplastic changes nor the presence of bacteria were observed at the mucosal level; only some parasitic ovoid structures on the edge of some areas of the gastric epithelium were formed. When performing Warthin–Starry staining, these unicellular parasites were also observed. The diagnosis showed presence of few ovoid parasitic structures in the gastric epithelium. In both groups of animals, esophagus did not show pathological changes.

## 4. Discussion

It is estimated that 50% of the world population has been infected by *H. pylori*, a disease that although in the early stages is not considered deadly, in the long term, it leads to more serious diseases, such as cancer. In recent years, infections related to bacteria and viruses have been associated with the development of gastric diseases including cancer, chronic gastritis, and MALT lymphoma. In particular, the role of *H. pylori* and Epstein–Barr virus (EBV) in gastric carcinogenesis has been evaluated. The relevance of the inflammatory response is hypothesized by recent studies showing how coinfection with *H. pylori* and EBV can cause tissue damage through inflammatory reactions or through increased contact between the CagA protein of *H. pylori* and EBV, which supports the increased activation of B cells in transit through the gastric mucosa [20]. Fasciana et al. demonstrated that the correlation of *H. pylori* and EBV is highly frequent [20]. Therefore, it is important to find new alternatives either for treatment or prophylaxis. The use of synthetic peptides designed from immunogenic proteins is considered an alternative for diagnosis and prophylaxis, but requires homogeneity in the antigenic preparation as described by Giammanco et al. [21]. In the present study, a murine model was used to determine the preventive potential of the MVTLINNE peptide, which is part of the terminal portion of a 52–55 kDa protein, identified as a homologue of citrate synthase, and has previously been described and patented for its usefulness as a diagnostic tool for *H. pylori* infection [22].

**Figure 4:**
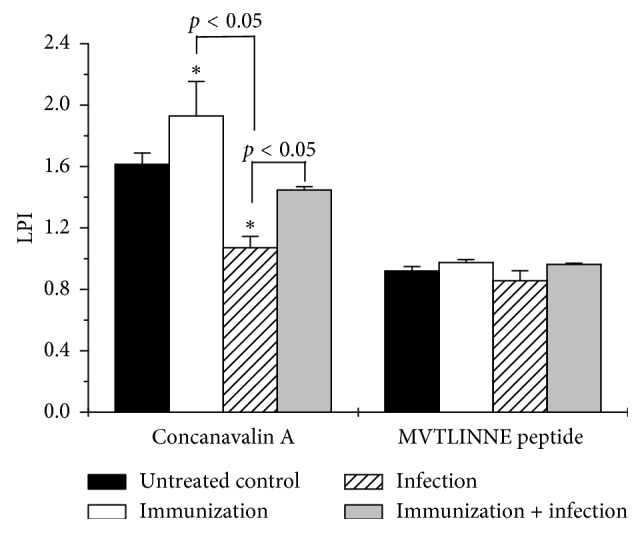
MVTLINNE peptide immunization stimulates Con A-mediated thymic lymphocyte proliferation. Lymphoproliferation was determined in thymus cell suspensions from immunized, immunized and infected, infected, and untreated control animals. Thymuses were surgically excised and mechanically dissociated into single-cell suspensions. Lymphocyte suspensions were then incubated in the presence or absence of Con A (6.25 *μ*g/ml) and/or MVTLINNE peptide (10 *μ*g/ml), and lymphoproliferation was measured by the MTT reduction assay, as explained in the text. Data represent LPI means ± SEM of triplicates from three independent experiments, *n*=5 in each group. Untreated control optical density was 0.48 ± 0.004. ^*∗*^*p* < 0.05, as compared with the untreated control.

In *H. pylori* preclinical models, the evaluation focuses on the proliferation of lesions in the stomach mucosa as a result of infection, and other methods such as molecular diagnosis are also useful in the evaluation of the success of an intervention therapy [23, 24]; however, its use requires expensive equipment and is not always available. Both the low mortality rate and the chronic nature of the disease limit preclinical models of the disease. Another important aspect in a preclinical model is the selection of the animal to be used; the most frequent is the mouse and in some studies the rat. However, the strain of animals used is also important when interpreting the studies, since there are important intrinsic physiological and immunological variations that can determine the treatment outcome. In the present study, an *in vivo* model of *H. pylori* was established in the BALB/c mouse; a noninvasive method of infection, administering the bacteria in the drinking water, was developed [17]. This model was used to evaluate the preventive efficacy of the administration of a synthetic peptide from a *H. pylori* protein; the MVTLINNE immunogenic peptide was administered twice, first intraperitoneally on week 1 and then subcutaneously as reinforcement; on week 4, in order to enhance the animal's immune response against infection. In this concern, the most used method for *in vivo* infection is the oral administration with a cannula of a known quantity of the bacteria [25]. However, this method requires considerable skill and carries risks for the welfare of the experimental animals; thus, an alternative method to reduce these risks was selected, in which bacteria are administered in the drinking water [17]. There is controversy regarding the time that the viability of the bacteria in water is maintained; however, several reports indicated that it is sufficient for oral infection [26, 27]. In our study, the data indicated that infected mice have immunological and histopathological response parameters consistent with infection (Figures 1–4).

Cellular immune response plays a critical role against *H. pylori* infection, which has been shown in immunodeficient mice models [28, 29]. In the present study, results showed that immunization with the MVTLINNE peptide stimulated the cellular immune response, as shown by the larger size of the spleen of immunized mice (Figure 1) and increased Con A-mediated proliferative response of thymus lymphocytes (Figure 4). *H. pylori*-mediated gastritis involves the release of cytokines from inflammatory cells, which contributes to maintain and amplify the local inflammation process. However, in this work, the analysis of TH1/TH2 cytokine profiles did not allow us to reach a conclusion about the success of the vaccination strategy, since no statistically significant differences were found between the experimental groups (Figure 2). However, there was an indication of an IL-6-mediated inflammatory response during infection and/or immunization (Figure 2). IL-6 plays an relevant role in innate and adaptive host defense by inducing IFN-*γ* production, immunoglobulin secretion, and neutrophil activation [30], and hence its involvement in protection against microbial infection *in vivo* [31]. In contrast, it was shown that IFN-*γ* may be involved in induction of *H. pylori*-mediated gastric inflammation [32].

It has been reported that *H. pylori* infection is associated with overexpression of IL-6 at the margin of gastric ulcer by macrophages [33, 34]. Furthermore, it was shown that gastric epithelium significantly contributed to the antral IL-1*β* and IL-6 response from *H. pylori*-infected duodenal ulcer patients and asymptomatic carriers [26]. In addition, increased production of IL-6 and TNF-*α* in human antral mucosa cultures from *H. pylori*-infected chronic gastritis patients has been observed by others [35].

Macrophage cytokine upregulation in gastric tissues during *H. pylori* infection has been proven [34], particularly increased expression levels of IL-1, TNF-*α*, and IL-6; IL-6 mRNA expression in gastritis tissues was shown to correlate with *H. pylori*-mediated infection and inflammation [34–37], and serum IL-6 concentrations were related to *H. pylori*-induced gastric cancer [38]. Since inflammation plays a significant role in gastric carcinogenesis, it has been suggested that polymorphisms in genes involved in inflammatory response may partly explain why only a subgroup of patients infected with *H. pylori* develop gastric cancer. Proinflammatory cytokine genetic background is believed to play a pathogenic role in age-related diseases; conversely, genetic variations determining increased production of anti-inflammatory cytokines or decreased production of proinflammatory cytokines have been shown to be associated with successful aging. It has been reported that polymorphisms in the IL-1 and IL-10 genes could contribute to determining the background for inflammation in which *H. pylori* infection might facilitate cancer development [39].

A potential mechanism by which *H. pylori* induces IL-6 production by macrophages in chronic gastritis patients was reported to be related to heat shock protein 60 stimulation [40]. Furthermore, in the present study, serum IL-17 was not altered by immunization and/or infection, although others have reported its upregulated expression in *H. pylori*-infected human gastric mucosa [41].

Because of the marginal efficacy and antibiotic resistance in the clinics and eradication of *H. pylori* protects from damaging gastric tissues, the development of a safe and effective vaccine for humans continues to be an active research issue [25]. The use of whole bacteria may be potentially harmful, whereas recombinant vaccines became an alternative for prophylaxis; however, additional immunogenic antigens must be tested [14]. In the present study, oral vaccination with the MVTLINNE peptide induced protective IgA and IgG antibodies, as shown in Table 1. Since *H. pylori* produces an intraluminal infection, immunity may be mediated, at least in part, by secretory IgA antibodies. For instance, human breast milk IgA protects children against *H. pylori* infection [42]. Oral immunization with killed *H. pylori* was reported to induce specific IgA and IgG antibodies in mice gastrointestinal secretions and sera [25]. It is recognized that oral vaccination induces an IgA-dependent mucosal immune response that eradicates long-term infection with *H. pylori* in mice [43, 44]. Oral administration of *H. pylori* recombinant urease plus adjuvant was reported to induce protective and long-lasting protective specific IgA immunity against challenge with virulent *H. felis* [15, 16, 45].

In our study, MVTLINNE peptide vaccination-mediated IgA production correlated with no alterations in the gastric mucosa and scarce presence of bacilli after *H. pylori* infection (Figure 3(b)), as compared with untreated control (Figure 3(a)). Taken together, these results indicated that prophylactic immunization significantly reduced the number of colonizing bacteria, which was associated with healthy gastric tissue [46].

## Figures and Tables

**Table 1:** Plasma immunoglobulins.

Experimental group	Antibody isotype
Untreated control	IgM
Immunized	IgM, IgA, IgG1, IgG2a
Infected	IgM
Immunized + infected	IgM, IgA, IgG1, IgG2a, IgG2b, IgG3

## Data Availability

The experimental data used to support the findings of this study are included within the article.

## Supplementary Materials

Supplementary Materials Spleen weights Student's *t*-test analysis: a Student's *t*-test analysis of spleen weights between immunized and control groups is depicted. One-way ANOVA test for peptide-immunization lymphoproliferation data: an ANOVA test for data related to the effect of peptide immunization on thymus lymphocyte proliferation, as compared with controls was performed. One-way ANOVA test for peptide-immunization and ConA-mediated lymphoproliferation data: an ANOVA test for data related to the effect of peptide immunization on concanavalin A-mediated thymus lymphocyte proliferation, as compared with controls, was performed. One-way ANOVA test for IL-6 production: an ANOVA test for data related to the effect of peptide immunization on IL-6 production, as compared with controls, was developed.
