# Supplementary material for: Immunization with a Synthetic Helicobacter pylori Peptide Induces Secretory IgA Antibodies and Protects Mice against Infection
Source: Can J Infect Dis Med Microbiol. 2019 Apr 1;2019:8595487. doi: 10.1155/2019/8595487 (PMC6466936; doi:10.1155/2019/8595487)
Supplement: Supplementary Materials — Spleen weights Student's t-test analysis: a Student's t-test analysis of spleen weights between immunized and control groups is depicted. One-way ANOVA test for peptide-immunization lymphoproliferation data: an ANOVA test for data related to the effect of peptide immunization on thymus lymphocyte proliferation, as compared with controls was performed. One-way ANOVA test for peptide-immunization and ConA-mediated lymphoproliferation data: an ANOVA test for data related to the effect of peptide immunization on concanavalin A-mediated thymus lymphocyte proliferation, as compared with controls, was performed. One-way ANOVA test for IL-6 production: an ANOVA test for data related to the effect of peptide immunization on IL-6 production, as compared with controls, was developed. [file 8595487.f1.zip › 8595487.f1/One-Way ANOVA-PROLIFERARION PEPTIDE ANALYSIS_CJIDMM_2669702.pdf]

One-Way Analysis of Variance for Independent or Correlated Samples

[Traducción en español]

The logic and computational details of the one-way ANOVA for independent and correlated samples are described in Chapters 13, 14, and 15 of Concepts and Applications.

Procedure:

- **Initial Setup:**T
  Enter the number of samples in your analysis (2, 3, 4, or 5) into the designated text field, then click the «Setup» button for either Independent Samples or Correlated Samples to indicate which version of the one-way ANOVA you wish to perform.T
- **Entering Data Directly into the Text Fields:**T
  After clicking the cursor into the scrollable text area for Sample 1, enter the values for that sample in sequence, pressing the carriage return key after each entry except the last. (On a Macintosh platform, the carriage return key is labeled 'Return'; on a Windows platform it is labeled 'Enter.') Perform the same procedure for the other samples in your analysis.T
- **Importing Data via Copy & Paste:**T
  Within the spreadsheet application or other source of your data, select and copy the column of data for sample 1. Then return to your web browser, click the cursor into the text area for sample 1 and perform the 'Paste' operation from the 'Edit' menu. Perform the same procedure for the other samples in your analysis.T
- **Data Check:**T
  For each sample, make sure that the final entry is **not** followed by a carriage return. (A carriage return after the final entry in a sample will be interpreted as an extra data entry whose value is zero. Importing data via the copy and paste procedure will almost always produce an extra carriage return at the end of a column.) After all values for a sample have been entered, click the cursor immediately to the right of the final entry in the list, then press the down-arrow key. If an extra line is present, the cursor will move downward. Extra lines can be removed by pressing the down arrow key until the cursor no longer moves, and then pressing the 'Backspace' key (on a Mac platform, 'delete') until the cursor stands immediately to the right of the final entry.T

  If you are performing a correlated-samples analysis, also make sure that the values for each sample are entered in the appropriate sequence. Note that a correlated-samples analysis presupposes equal numbers of observations for each sample in the analysis.T
- **When all** sample values have been entered, click the button labeled «Calculate.» For independent samples the default analysis is a standard weighted- means analysis. If you wish to perform an unweighted- means analysis, click the «Unweighted» button before calculating.

Note that when the number of samples is **k**=2, the analysis of variance (standard weighted-means analysis) is equivalent to a non-directional **t**-test with **F**=**t**^{2}.

Setup

Number of samples in analysis =	5
Independent Samples<br>Correlated Samples	Independent Samples k=5<br>standard weighted-means analysis
Unweighted	Click this button only if you wish to perform an unweighted-means analysis. Advice: do not perform an unweighted-means analysis unless you have a clear reason for doing so.
Weighted	Click this button to return to a standard weighted-means analysis

Data Entry	Inmunizado Infectado	Inmunizado	Control	Control infectado
Sample 1	Sample 2	Sample 3	Sample 4	Sample 5
1<br>1<br>1<br>1<br>1	0.937955394<br>0.960928644<br>0.956910245<br>0.983280509<br>0.97817901	0.989480749<br>0.940479387<br>1.043963982<br>0.96763189<br>0.938502126	0.905163797<br>0.884666627<br>0.848422572<br>0.950093108<br>1.009909172	0.746975153<br>0.741062731<br>1.008801863<br>0.768038953<br>1.017942494

Reset Calculate

Data Summary

	Samples					
	1	2	3	4	5	Total
N	5	5	5	5	5	25
-∑X	5	4.817254	4.880058	4.598255	4.282821	23.578388
-Mean	1	0.963451	0.976012	0.919651	0.856564	0.943136
-∑X^2	5	4.642496	4.770532	4.244371	3.750918	22.408317
Variance	0	0.000327	0.001885	0.003895	0.020602	0.007113
Std.Dev.	0	0.018092	0.043413	0.062411	0.143533	0.084336
Std.Err.	0	0.008091	0.019415	0.027911	0.06419	0.016867

standard weighted-means analysis

ANOVA Summary Independent Samples k=[illegible]

Source	SS	df	MS	F	P
Treatment [between groups]	0.063866	4	0.015967	2.99	0.043673
Error	0.106835	20	0.005342		
Ss/Bl					Graph Maker
Total	0.170701	24			

Ss/Bl = Subjects or Blocks depending on the design.
Applicable only to correlated-samples ANOVA.

Tukey HSD Test

HSD[.05]=0.14; HSD[.01]=0.17	
M1 vs M2	nonsignificant
M1 vs M3	nonsignificant
M1 vs M4	nonsignificant
M1 vs M5	P<.05
M2 vs M3	nonsignificant
M2 vs M4	nonsignificant
M2 vs M5	nonsignificant
M3 vs M4	nonsignificant
M3 vs M5	nonsignificant
M4 vs M5	nonsignificant

M1 = mean of Sample 1
M2 = mean of Sample 2
and so forth.

HSD = the absolute [unsigned] difference between any two sample means required for significance at the designated level. HSD[.05] for the .05 level; HSD[.01] for the .01 level.

Print this Page

**Home**	Click this link **only** if you did not arrive here via the VassarStats main page.

©Richard Lowry 2001-2016
All rights reserved.
