# Supplementary material for: Immunization with a Synthetic Helicobacter pylori Peptide Induces Secretory IgA Antibodies and Protects Mice against Infection
Source: Can J Infect Dis Med Microbiol. 2019 Apr 1;2019:8595487. doi: 10.1155/2019/8595487 (PMC6466936; doi:10.1155/2019/8595487)
Supplement: Supplementary Materials — Spleen weights Student's t-test analysis: a Student's t-test analysis of spleen weights between immunized and control groups is depicted. One-way ANOVA test for peptide-immunization lymphoproliferation data: an ANOVA test for data related to the effect of peptide immunization on thymus lymphocyte proliferation, as compared with controls was performed. One-way ANOVA test for peptide-immunization and ConA-mediated lymphoproliferation data: an ANOVA test for data related to the effect of peptide immunization on concanavalin A-mediated thymus lymphocyte proliferation, as compared with controls, was performed. One-way ANOVA test for IL-6 production: an ANOVA test for data related to the effect of peptide immunization on IL-6 production, as compared with controls, was developed. [file 8595487.f1.zip › 8595487.f1/Spleen Weight t test analysis_CJIDMM_2669701.pdf]

t-Test for Independent or Correlated Samples

[Traducción en español]

The logic and computational details of two-sample t-tests are described in Chapters 9-12 of the online text *Concepts & Applications of Inferential Statistics*. For the independent-samples t-test, this unit will perform both the "usual" t-test, which assumes that the two samples have equal variances, and the alternative t-test, which assumes that the two samples have *un*equal variances. (A good formulaic summary of the unequal-variances t-test can be found on the StatsDirect web site. A more thorough account appears in the online journal *Behavioral Ecology*.)

Setup

Independent Samples

Correlated Samples

Independent Samples

Data Entry

Sample A	Sample B
161.8<br>170.2<br>139.2<br>110.3<br>110<br><br>inmunizado	98.3<br>101.8<br>103.1<br>101.2<br>99.8<br><br>control

Please be sure to perform the Data Check procedure.

Reset Calculate

Procedure

followed by a carriage return. (A carriage return after the final entry in a sample will be interpreted as an extra data entry whose value is zero. Importing data via the copy and paste procedure will almost always produce an extra carriage return at the end of a column.) After all values for a sample have been entered, click the cursor immediately to the right of the final entry in the list, then press the down-arrow key. If an extra line is present, the cursor will move downward. Extra lines can be removed by pressing the down-arrow key until the cursor no longer moves, and then pressing the 'Backspace' key (on a Mac platform, 'delete') until the cursor stands immediately to the right of the final entry.

If you are performing a correlated-samples analysis, also make sure that the values for each sample are entered in the appropriate sequence. Note that a correlated-samples analysis presupposes equal numbers of observations for each sample.

When all sample values have been entered, click the button labeled 'Calculate.'

Data Summary

	A	B	Total
n	5	5	10
ΣX			

$\sum X$	691.5	504.2	1195.7
- $\sum X^2$	98790.01	50857.22	149647.229
SS	3155.56	13.692	6677.381
mean	138.3	100.84	119.57

---

*Results*Q

$Mean_a - Mean_b$	t	df
37.46	+2.98	8

P		
	one-tailed	0.0088
	two-tailed	0.017600

For independent samples, these results pertain to the "usual" t-test, which assumes that the two samples have equal variances.

---

*F-Test for the Significance of the Difference between the Variances of the Two Samples*Q

df_1	df_2	F	P
4	4	230.47	<.0001

[Applicable only to independent samples.]
P>.05 indicates no significant difference detected between the variances of the two samples.

---

t-Test Assuming Unequal Sample Variances
*[Applicable only to independent samples.]*Q

$Mean_a - Mean_b$	t	df
37.46	2.98	4.03

P		
	one-tailed	0.020454
	two-tailed	0.040908

		Confidence Intervals	
	Observed	0.95	0.99
$Mean_a$	138.3	± 34.9195	± 57.7805
$Mean_b$	100.84	± 2.3002	± 3.8061
$Mean_a - Mean_b$ [Assuming equal sample variances.]	37.46	± 29.0787	± 42.2963
$Mean_a - Mean_b$ [Assuming unequal sample variances.]	37.46	± 34.9952	± 57.9057
	Independent Samples		

For purposes of significance tests and calculation of confidence intervals, values of df associated with the unequal-variance condition are rounded to the nearest integer.

	[illegible]

Print this Window

**Home**	Click this link **only** if you did not arrive here via the VassarStats main page.

©Richard Lowry 2001-2019
All rights reserved.
